# Extreme longevity variants at the *FOXO3* locus may moderate *FOXO3* isoform levels

**DOI:** 10.1007/s11357-021-00431-0

**Published:** 2021-08-26

**Authors:** Ryan Frankum, Tom S. O. Jameson, Bridget A. Knight, Francis B. Stephens, Benjamin T. Wall, Timothy A. Donlon, Trevor Torigoe, Bradley J. Willcox, D. Craig Willcox, Richard C. Allsopp, Lorna W. Harries

**Affiliations:** 1 Institute of Biomedical and Clinical Science, University of Exeter Medical School, Barrack Road, Exeter, EX2 5DW UK; 2 College of Life and Environmental Sciences, University of Exeter, Exeter, UK; 3 NIHR Exeter Clinical Research Facility, Royal Devon and Exeter NHS Foundation Trust, Exeter, UK; 4 Honolulu Heart Program (HHP)/Honolulu-Asia Aging Study (HAAS), Department of Research, Kuakini Medical Center, Honolulu, HI 96817 USA; 5 Departments of Cell & Molecular Biology and Pathology, University of Hawaii, Honolulu, HI 96813 USA; 6 Institute for Biogenesis Research, Department of Anatomy, Biochemistry and Physiology, John A. Burns School of Medicine, University of Hawaii, Honolulu, HI USA; 7 Department of Geriatric Medicine, John A. Burns School of Medicine, University of Hawaii, Honolulu, HI 96817 USA; 8 Department of Research, Kuakini Medical Center, Honolulu, HI 96817 USA; 9 Okinawa International University, Okinawa, Japan

**Keywords:** *FOXO3*, Longevity, Human, Variation, Isoforms

## Abstract

The rs2802292, rs2764264 and rs13217795 variants of *FOXO3* have been associated with extreme longevity in multiple human populations, but the mechanisms underpinning this remain unclear. We aimed to characterise potential effects of longevity-associated variation on the expression and mRNA processing of the *FOXO3* gene. We performed a comprehensive assessment of *FOXO3* isoform usage across a wide variety of human tissues and carried out a bioinformatic analysis of the potential for longevity-associated variants to disrupt regulatory regions involved in isoform choice. We then related the expression of full length and 5′ truncated *FOXO3* isoforms to rs13217795 genotype in peripheral blood and skeletal muscle from individuals of different rs13217795 genotypes. *FOXO3* isoforms displayed considerable tissue specificity. We determined that rs13231195 and its tightly aligned proxy variant rs9400239 may lie in regulatory regions involved in isoform choice. The longevity allele at rs13217795 was associated with increased levels of full length *FOXO3* isoforms in peripheral blood and a decrease in truncated *FOXO3* isoforms in skeletal muscle RNA. We suggest that the longevity effect of *FOXO3* SNPs may in part derive from a shift in isoform usage in skeletal muscle away from the production of 5′ truncated *FOXO3* isoforms lacking a complete forkhead DNA binding domain, which may have compromised functionality.

**Supplementary Information:**

The online version contains supplementary material available at 10.1007/s11357-021-00431-0.

## Introduction

Human ageing is highly heterogeneous. Some people reach old age with good maintained health and quality of life, whilst others succumb to age-related diseases whilst still relatively young. The molecular basis behind these differences is still unclear. Long life often goes hand-in-hand with avoidance of age-related disease, in a phenomenon termed compression of morbidity [12]. This suggests that the factors that govern lifespan also govern ‘health span’. Although environment and lifestyle contribute the largest component to successful ageing, genetic influences are also apparent. All-cause mortality was seen to decrease by 19% and 14% for the children of long-lived mothers and long-lived fathers respectively, compared to their cohabiting spouses [8]. Offspring of long-lived parents demonstrated lower incidence of cancer, diabetes, heart disease and stroke [8] and were also demonstrated to demonstrate lower rates of age-related cognitive decline [9]. The heritability of longevity is reported to be ~ 16% [19]. These observations suggest that some people are genetically predisposed to a long life.

Several genetic variants associated with longevity have been reported. A large meta-analyses of associations with parental lifespan carried out in 1 million participants reported associations with several loci including those containing the *CDKN2B-AS1, ATXN2/BRAP, FURIN/FES, ZW10, PSORS1C3, ABO, ZC3HC1, IGF2R EBF1* and *FOXO3* genes [30]. *FOXO3* (Forkhead Box O3) is one of four FOXO human homologues of *Daf16*, the first gene to be associated with lifespan in the nematode *C. elegans* [20]. It is a multifunctional transcriptional regulator with roles in multiple processes such as apoptosis, insulin signalling, metabolism, immunity and inflammation, proteostasis and autophagy, response to cellular stress and control of cellular differentiation [26]. Associations between genetic variation within or near the *FOXO3* gene and longevity were first reported in a population of male American individuals of Japanese ancestry, which provided evidence for an association between rs2802292, rs2764264 and rs13217795 and extreme longevity [31]. These findings have now been replicated in 11 different studies in populations of diverse ancestries [26]. The strongest association reported is with variant rs2802292, located in intron 2 of the *FOXO3* gene, which confers an odds ratio for extreme longevity of 2.75 and is also linked with biological markers of successful ageing and lower incidence of age-related disease [31]. This variant has also been linked with preservation of telomere length in the peripheral blood of individuals carrying longevity alleles at these loci [4].

Genetic variation can affect gene expression or activity by many mechanisms. Coding variants can change the amino acid sequence of the peptides produced, but the vast majority of variation associated with longevity, like most other traits, maps to non-coding regions of the genome [14]. Non-coding regions such as the 5′ and 3′ untranslated regions (UTRs), along with introns and intergenic regions often contain regulatory elements such as enhancers or sites of posttranscriptional modification. The *FOXO3* longevity-associated variants are primarily located in or near intron 2 and do not alter the coding sequences of the gene. The underlying mechanism by which these variants lead to extreme longevity remains to be elucidated, although a search for linked functional variants indicated 13 putative regulatory SNPs that significantly modified 18 transcription factor/enhancer binding sites in or near *FOXO3* [7]. Intronic variation is also commonly associated with alterations in alternative isoform usage, by virtue of disruption of splicing regulatory elements such as intron splicing enhancer or silencer elements [1]. ENSEMBL indicates that human *FOXO3* gene encodes three protein-coding alternatively expressed isoforms with differences in upstream exon usage (ENST00000343882.10, ENST00000406360.2 and ENST00000540898.1), although these are poorly characterised and little studied.

Here, we aimed to (i) characterise an expression profile for alternatively-expressed *FOXO3* isoforms in a panel of human tissues, (ii) to map the rs2802292, rs2764264 and rs13217795 variants of *FOXO3* (and their proxies) to potential splicing or expression regulatory elements that may control *FOXO3* isoform expression and (iii) to relate the expression of variants located in potential isoform regulatory elements to *FOXO3* genotype in human peripheral blood and skeletal muscle. We confirmed the presence of two classes of *FOXO3* isoforms: long (*FOXO3-FL*) and short (*FOXO3-TR*), and determined that whilst *FOXO3-FL* isoforms are present in all most *FOXO3*-expressing tissues, the profile of *FOXO3-TR* is more restricted. We determined that variant rs13217795 lies in a region of the genome with characteristics of an alternative promoter for *FOXO3* with potential to regulate *FOXO3-TR*, and that genotype at this variant is associated with increased *FOXO3-FL* expression in peripheral blood and with decreased *FOXO3-TR* expression in human skeletal muscle. *FOXO3-TR* is predicted to encode a truncated FOXO3 protein, which lacks part of the forkhead domain and several important points of posttranslational modification. We propose that the longevity effect conferred by rs13217795 may arise from a change in the balance of *FOXO3-FL* and *FOXO3-TR* isoforms towards a decrease in the production of the *FOXO3-TR,* which is predicted to lack DNA binding and to have lower activity.

## Methods

### Design of qRT-PCR probes to long and short FOXO3 isoforms

**Fig. 1:**
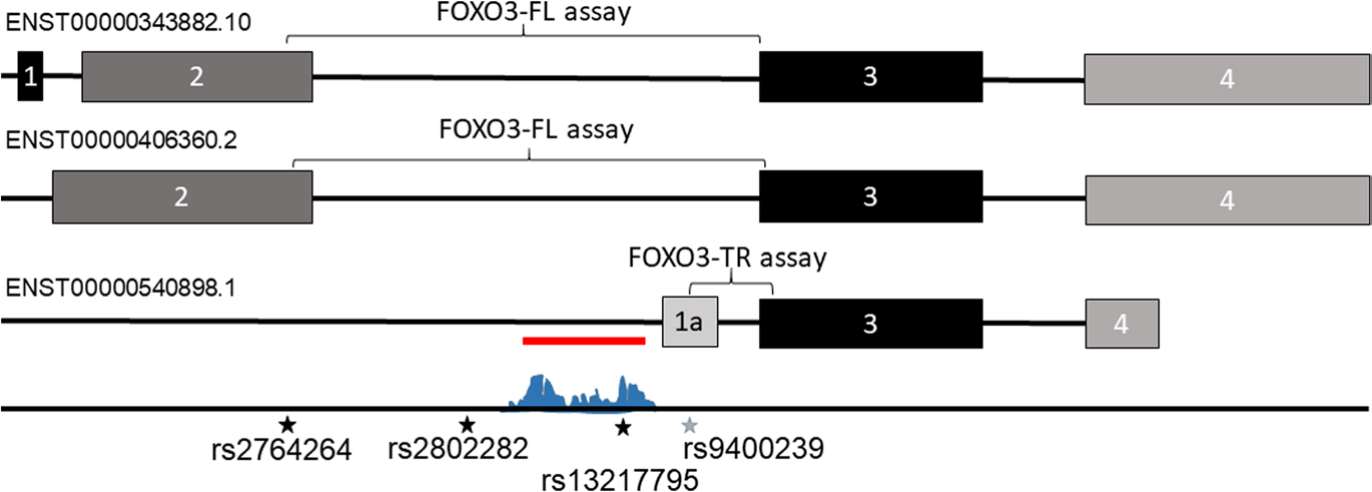
The structure of *FOXO3* isoforms. The structure of the exon structure and potential regulatory regions of the FOXO3 gene are given here. Exons are given by black or grey boxes, and introns by a black line. The position of the FOXO3-FL and FOXO3-TR assays that span the exon 2–3 and exon 1a-3 splice junctions respectively are indicated. The potential promoter is indicated by the red line and the presence of a layered histone modification peak indicated in blue, as observed using https://www.genome.ucsc.edu/. The positions of the extreme longevity index SNPs rs2802292, rs2764264 and rs13217795 are given by a black star, and the rs9400239 proxy variant of rs13217795 is given by a grey star are indicated

The sequences of *FOXO3* transcripts corresponding to long and short *FOXO3* isoforms were accessed from the ENSEMBL genome browser (https://www.ensembl.org), and assays specific to the two full length isoforms (*FOXO3-FL*; ENST00000343882.10 and ENST00000406360.2) or the truncated *FOXO3* isoform (*FOXO3-TR*; ENST00000540898.1) were designed. The *FOXO3-FL* assay was targeted over the exon 2/exon 3 boundary shared by both full length isoforms and thus did not differentiate between them. The *FOXO3-TR* assay was targeted over the exon 1a/exon 3 boundary and was specific to ENST00000540898.1 (Fig. 1). Probes and primers were as follows: *FOXO3-FL*; forward primer — GGATAAGGGCGACAGCAACA, a reverse primer —GAATCGACTATGCAGTGACAGGTT and probe — FAM-CCGGATGGAGTTCTTC-MBG. *FOXO3-TR*; forward primer — GCCCTGGAACCTTTTGGCTTAA, reverse primer — CGACTATGCAGTGACAGGTTGT and probe FAM–CTCGGAAAACAAACTCC-MGB. Assays were purchased from Thermo Fisher (Waltham, USA).

### Validation of FOXO3 isoform-specific assays

The performance of *FOXO3-FL* and *FOXO3-TR* assays was assessed using standard curve analysis. One hundred nanograms of skeletal muscle total RNA was reverse transcribed for each sample in a total volume of 20µl using the Evoscript cDNA synthesis kit (Roche, Burgess Hill, UK) according to the manufacturer’s instructions. cDNA then underwent seven 1:2 serial dilutions to establish accuracy, sensitivity and linear range. Reactions contained 2.5 µl TaqMan Universal Mastermix II (Thermo Fisher, Waltham, USA) 0.9µM each primer, 0.25µM probe (Thermo Fisher, Waltham, USA), 1.25µl H_2_O and 1µl cDNA in a total volume of 5µl. Cycling conditions were 95 ^O^C for 10 min prior to cycling, then 95 ^O^C for 15 s and 60 ^O^C for a minute for 40 cycles.

### Characterisation of the expression profile of FOXO3-FL and FOXO3-TR in human tissues

Samples from different human tissues (skeletal muscle, pancreas, lung, pons, ovary, oesophagus, adipose, bladder, thyroid, breast, testes, adrenal gland, small intestine, thymus, trachea, cervix, prostate, medulla oblongata, colon, stomach, postcentral gyrus, kidney, hippocampus, spleen, salivary gland, parietal lobe, liver, cerebellum, heart, cerebral cortex, placenta, temporal lobe, uterus, total brain, pituitary, bone marrow, pooled islets and blood) were obtained from commercially-sourced, tissue-specific RNA panels (Becton, Dickinson & Co., Franklin Lakes, NJ, USA: BioChain Institute, Newark, CA, USA: Ambion®, Austin, TX, USA). One hundred nanograms of total RNA was reverse transcribed for each sample in a total volume of 20µl using the Evoscript cDNA synthesis kit (Roche, Burgess Hill, UK) according to the manufacturer’s instructions. PCR reactions contained 2.5µl TaqMan Universal Mastermix (no AMPerase) (Applied Biosystems, Foster City, USA), 0.9µM each primer, 0.25µM probe, 1.25µl H_2_O and 1µl cDNA reverse transcribed as above in a total volume of 5µl. PCR conditions were a single cycle of 95ºC for 10 min followed by 40 cycles of 95 °C for 15 s and 60 °C for 1 min. The amount of *FOXO3-FL* and *FOXO3-TR* isoforms in each tissue sample was then quantified by isoform-specific qRT-PCR by the Comparative Ct method relative to the geometric mean of Beta 2 Microglobulin (*B2M*), Glyceraldehyde 3-phosphate dehydrogenase (*GAPDH*) and beta glucoronidase (*GUSB*) endogenous control gene expression, with adjustment for assay efficiencies. Expression levels were then normalised to the average level of *FOXO3-FL* isoforms over the entire tissue panel.

### Bioinformatic analysis of potential FOXO3 regulatory regions

To determine for rs2802292, rs2764264 and rs13217795 to interfere with splicing or expression of the *FOXO3* gene, we firstly determined all the proxy SNPS that were in complete linkage disequilibrium (*D*’ =  > 0.95, *r*^2^ > 0.80) with these index SNPs using SNPsnap (https://data.broadinstitute.org/mpg/snpsnap/about.html). Index SNPs and proxies were then mapped to the known regulatory regions of the *FOXO3* gene with reference to the UCSC genome browser (https://www.genome.ucsc.edu/). SNPs were designated for follow-up if they were located within a predicted promoter element or within 50 bp of a splice site.

### Profiling of FOXO3 isoforms in peripheral blood samples

We next profiled *FOXO3* isoform levels in RNA extracted from 51 previously genotyped peripheral blood samples from the Exeter 10,000/Peninsula Research Bank study (https://exetercrfnihr.org/about/exeter-10000-prb/) selected on the basis of rs13217795 genotype. This collection is a cross sectional population study consisting of samples collected from volunteer individuals living in the South West of England and recruited since 2010. Whole blood samples were collected in 2011/2012 using the PAXgene system [5] and extracted using the PAXgene Blood RNA kit (Qiagen, Paisley, UK). Access to these anonymised samples was approved by the ethically approved Peninsula Research Bank Steering Committee (14/SW/1089). Our sample set consisted of 18 individuals homozygous for the major allele of rs13217795 (TT), 17 samples heterozygous for rs13217795 (CT) and 17 samples homozygous for the longevity allele of rs13217795 (CC). Participant characteristics are given in Table 1. Levels of *FOXO3-FL* and *FOXO3-TR* isoforms were then quantified relative to the geometric mean of the B2M and GAPDH genes by qRT-PCR as described above, with adjustment for assay efficiencies. Data were then normalised to the mean level of the full-length isoforms in the rs13217795 heterozygous samples. Statistical significance was then determined by ANOVA comparing age, sex and body mass index (BMI) covariates by genotype. Significant covariates were taken into account within a univariate linear model using the IBM SPSS Statistics 26 program (IBM SPSS version 26 release 26.0.0.0, Armonk, NY).

**Table 1:** Table of participant details. Patient metrics for peripheral blood and skeletal muscle samples, displaying the average age, sex and BMI with SD and number of samples for each genotype. *P* values indicate one-way ANOVA between genotypes by variable

**Peripheral blood samples**				
	**rs13217795** **major homozygotes (TT) (*n* = 18)**	**rs13217795** **heterozygotes (CT) (*n* = 17)**	**rs13217795** **minor homozygotes (CC) (*n* = 17)**	***p* value**
Mean age	49.94 (15.85)	53.75 (9.35)	51.24 (15.758)	0.738
Sex (% female)	81	44	65	0.090
Mean BMI	25.31 (5.29)	27.62 (3.79)	25.69 (4.95)	0.341
**Skeletal muscle samples**				
	**rs13217795** **major homozygotes (TT) (*n* = 27)**	**rs13217795** **heterozygotes (CT) (*n* = 25)**	**rs13217795** **minor homozygotes (CC) (*n* = 8)**	***p* value**
Mean age	22.37 (3.47)	20.96 (3.09)	23 (4.34)	0.212
Sex (% female)	30	16	12.5	0.404
Mean BMI	23.06 (3.48)	24.56 (3.94)	25.45 (2.52)	0.160

### Characterisation of the expression profile of FOXO3-FL and FOXO3-TR in human skeletal muscle

In total, 64 human skeletal muscle samples were obtained from previously published studies of human muscle metabolism [6, 18, 21, 29]. Four samples were omitted due to unsuccessful genotyping. All volunteers abstained from alcohol and strenuous exercise for at least 48 h prior to the sample obtained. Samples were obtained from the vastus lateralis muscle and were dissected free of visible connective tissue, fat and blood immediately prior to snap freezing. Patient characteristics are given in Table 1. RNA was extracted from 20 mg homogenised muscle sample using TRIzol reagent (Thermo Fisher, Waltham, USA). RNA concentration was quantified by ND-2000 Nanodrop Spectrophotometer (NanoDrop,Thermo Fisher, Waltham City, USA). The genotype of each sample was determined by qPCR from the presence of heterogeneous RNA (hnRNA) in the RNA samples using 2.5 µl TaqMan Universal Mastermix II (Thermo Fisher, Waltham City, USA), 0.9µM of each primer and 0.2µM of each probe (genotyping assay ID = C_9174543_10) and 1 µl RNA in a total volume of 5 µl. Cycling conditions were 60 °C for 30 s, followed by 95 °C for 10 min prior to cycling. Samples were then cycled between 95 °C for 15 s and 60 °C for a minute for 40 cycles. Following this, samples were held at 60 °C for 30 s. *FOXO3* isoform levels were quantified by qRT-PCR as described above. Levels of *FOXO3-FL* and *FOXO3-TR* isoforms were then quantified relative to the geometric mean of the *B2M* and *GAPDH* genes by qRT-PCR as described above, with adjustment for assay efficiencies. *GUSB* was omitted from this analysis as it proved unstable baseline in our sample set. Data were then normalised to the mean level of the full length isoforms in the rs13217795 heterozygous samples. Statistical significance was then determined by ANOVA comparing age, sex and body mass index (BMI) covariates by genotype. Significant covariates were taken into account within a univariate linear model using the IBM SPSS Statistics 26 program (IBM SPSS version 26 release 26.0.0.0, Armonk, NY).

### Assessment of FOXO3-FL isoform expression by age in human peripheral blood

To determine whether *FOXO3* isoform expression alters with age, we carried out an assessment of the association between age and *FOXO3-FL* isoform expression exclusively in the 16 individuals homozygous for the major (T/T) allele, to negate the effect of genotype by linear regression analysis. We were unfortunately unable to assess the effect of age on the truncated FOXO3-TR isoform since this isoform was only expressed in skeletal muscle, and the age range of the donors of our muscle samples was very limited (18 to 31 years).

## Results

### Validation of FOXO3-FL and FOXO3-TR assays

Quantitative real-time PCR assays were designed to *FOXO3* full length (*FOXO3-FL*) and truncated (*FOXO3-TR*) isoforms and validated by standard curve analysis to determine accuracy, efficiency and linear range. *FOXO3-FL* and *FOXO3-TR* assays proved sensitive and accurate over a dynamic linear range of serial dilutions, with gradients of − 3.774 and − 3.271 and *R*^2^ values between replicates of 0.954 and 0.933 respectively. Representative standard curves for FOXO3-FL and FOXO3-TR isoforms are given in supplementary figure S1.

### Expression pattern of FOXO3-FL and FOXO3-TR isoforms

**Fig. 2:**
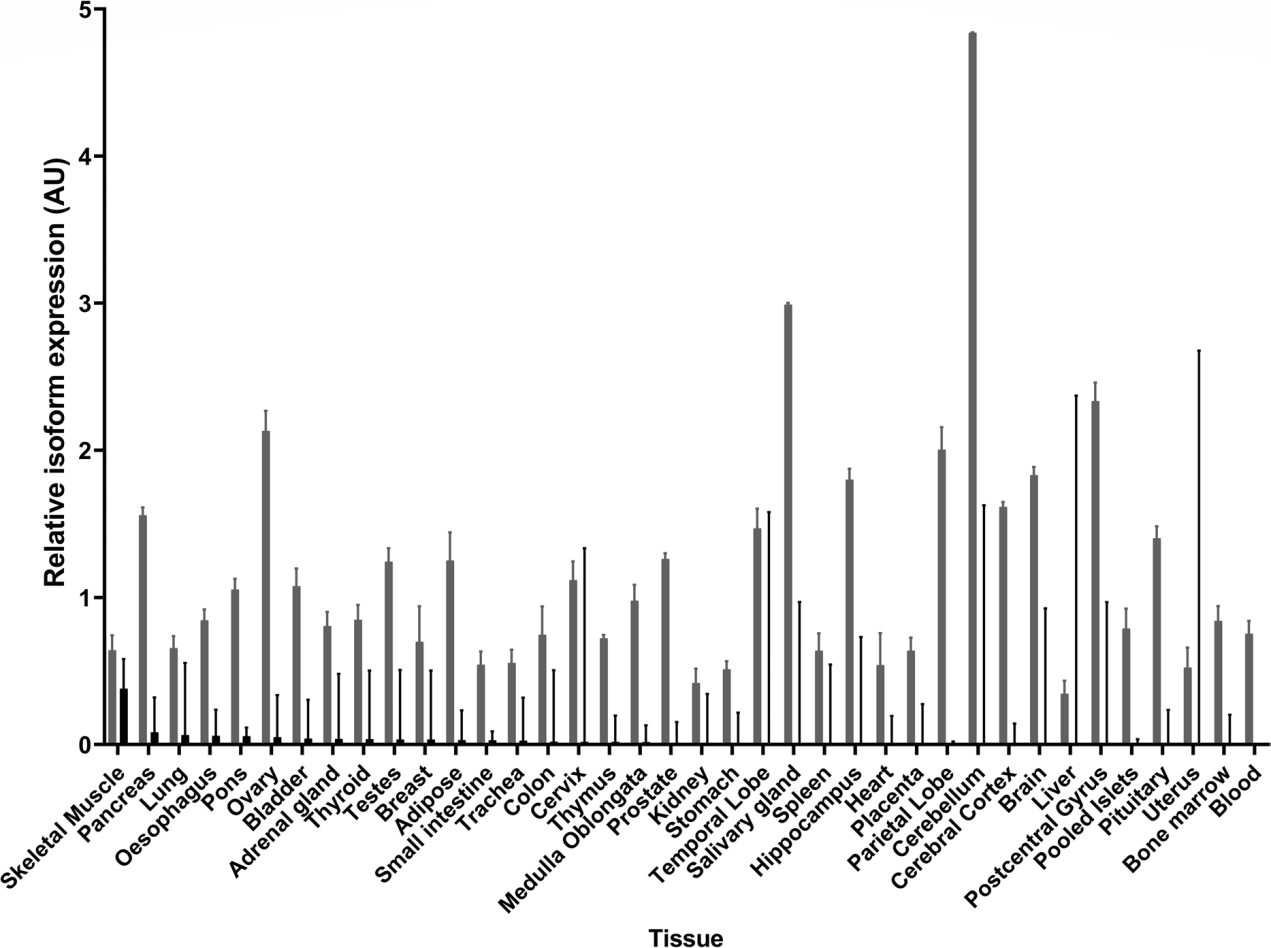
The expression of FOXO3 isoforms across multiple tissues. The figure gives the expression pattern of full length and truncated FOXO3 isoforms across an extended panel of human tissues. Mean relative mRNA expression in arbitrary units (AU) is given on the *Y* axis, whilst tissue identity is given on the *X* axis. Full length isoforms ENST00000343882.10 and ENTS0000406360.2 (FOXO3-FL) are given by light grey bars and the truncated isoform ENST00000540898.1 (FOXO3-TR) is given by black bars. Error bars represent standard deviation of quantification (SD)

The expression of *FOXO3-FL* and *FOXO3-TR* were determined over 38 commercially sourced different human tissues (Becton, Dickinson & Co., Franklin Lakes, NJ, USA: BioChain Institute, Newark, CA, USA: Ambion®, Austin, TX, USA). All tissues except blood were found to express both *FOXO3-TR* and *FOXO3-FL*; however, *FOXO3-TR* was expressed at a reduced level in all tissues compared to *FOXO3-FL*. The expression of *FOXO3-FL* compared to *FOXO3-TR* was highly variable by tissue type, with skeletal muscle expressing the highest level of *FOXO3-TR* to *FOXO3-FL* across the panel. *FOXO3-FL* isoforms were highly expressed in most brain regions (postcentral gyrus, hippocampus, parietal lobe, cerebellum, cerebral cortex and temporal lobe) as well as in salivary and pituitary glands and whole blood. Levels of *FOXO3-TR* were highest in skeletal muscle, followed by many tissues of the endocrine, digestive or respiratory systems (pancreas, ovary, oesophagus, adrenal gland, adipose, testes, small intestine, thymus, trachea and lung) (Fig. 2).

### Bioinformatic evaluation of FOXO3 SNPs for potential regulatory effects

We next assessed the potential for *FOXO3* rs2802292, rs2764264 and rs13217795 SNPs and their tightly linked proxies to disrupt predicted regulatory regions. Variant rs2802292 had seven proxies in almost complete linkage disequilibrium (LD) (*D*’ of 0.99 and *r*^2^ from 0.89 to 0.99; Table 2). None of these variants, or rs2802292 itself, was located within 50 bp of a splice site or within a predicted promoter sequence (Table 2). We identified no proxies in complete LD with rs2764264 and rs2764264 did not map close to any splice site or predicted regulatory region (Table 2). Conversely, rs13217795 demonstrated seven proxy SNPs in tight LD (*D*’ = 0.96 to 0.99; *r*^2^ = 0.84–0.95). Of these, although none mapped in proximity to a splice site, rs9400239 mapped to the alternative non-coding exon 1a of *FOXO3-TR*, and rs13217795 itself mapped to a predicted regulatory region for an alternative promoter with potential to regulate the expression of *FOXO3-TR* (Table 2; Fig. 1). As rs9400239 and rs13217795 are in almost complete LD, we elected to take forward rs13217795 for analysis on the basis that it will capture both SNPs.

**Table 2:** Potential regulatory effects of longevity-associated variants at *FOXO3* and their proxies. We identified proxies for the three longevity-associated *FOXO3* variants and mapped these to potential regulatory regions in or around the *FOXO3* gene as described in (https://www.genome.ucsc.edu/, accessed 20.01.21). Index SNPS are indicated in bold type, and those mapping to potential regulatory regions in italicised underlined type. Linkage disequilibrium is given by measured of *D*’ and *r*^2^

	*D*’	*r*^2^	Location	< 50 bp from splice site?	In predicted promoter?
**rs2802292**	**-**	**-**	**Intron 2**	**No**	**No**
rs2253310	0.99	0.99	Intron 2	No	No
rs2764261	0.99	0.88	Intron 2	No	No
rs2802295	0.99	0.98	Intron 2	No	No
rs2764265	0.99	0.89	Intron 2	No	No
rs2490272	0.99	0.94	Intron 2	No	No
rs2802288	0.99	0.99	Intron 2	No	No
rs2802290	0.99	0.99	Intron 2	No	No
**rs2764264**	**-**	**-**	**Intron 2**	**No**	**No**
***rs13217795***	-	-	***Intron 2***	***No***	***Yes***
rs71015554	0.99	0.84	Intron 2	No	No
rs9398171	0.97	0.93	Intron 2	No	No
*rs9400239*	*0.99*	*0.95*	*Exon 1a*	*No*	*No*
rs4946932	0.99	0.95	Intron 2	No	No
rs10457180	0.99	0.86	Intron 2	No	No
rs2022464	0.99	0.94	Intron 2	No	No
rs138068040	0.96	0.91	Intron 2	No	No

### The longevity-associated C allele of rs13217795 is associated with elevated expression of FOXO3-FL isoforms in peripheral blood

**Fig. 3:**
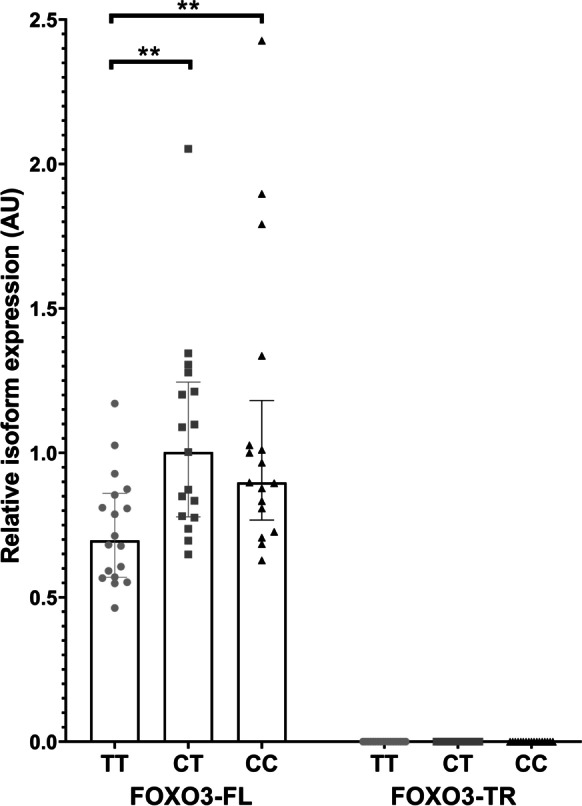
Relationship between *FOXO3* genotype and *FOXO3* isoform expression in peripheral blood RNA samples. The relationship between *FOXO3* full length and truncated isoform levels and genotype at rs13217795 is given for peripheral blood samples originating from the Exeter 10 K study. Isoform identity and genotype are given on the *X* axis, and median relative expression in arbitrary units (AU) is given on the *Y* axis. Statistical significance of logged data is indicated by stars; *p ≤ 0.05, **p ≤ 0.01, ***p ≤ 0.001. Circles indicate major allele (T) homozygotes, squares indicate heterozygous samples (C/T) and triangles indicate minor allele (C) homozygotes. Error bars represent interquartile range. No expression of *FOXO3-TR* isoform was detected in peripheral blood

We measured levels of *FOXO3-FL* and *FOXO3-TR* isoforms in RNA from human peripheral blood from the Exeter 10 K study, and related these to genotype at rs13217795. We identified that increased expression of the *FOXO3-FL* isoforms was associated with the extreme longevity ‘C’ allele, and that effects were apparent in both heterozygotes and longevity allele homozygotes, with effects demonstrating a dominant pattern, with no difference between individuals carrying either one or two C alleles (Fig. 3). In heterozygous individuals (‘C/T’), the logged mean expression of *FOXO3-FL* isoforms was 1.05 (SD = 0.33) compared with − 0.73 (SD = 0.19) in individuals homozygous for the ‘T’ allele (*p* = 0.004). In individuals carrying two copies of the longevity ‘C’ allele, logged mean expression was 1.1 (SD = 0.40) compared with 0.73 (SD = 0.249) in individuals homozygous for the major ‘T’ allele (*p* = 0.003). We detected no expression of *FOXO3-TR* isoforms in peripheral blood, consistent with our findings in the tissue panel (Fig. 2).

### The longevity-associated C allele of rs13217795 is associated with decreased expression of FOXO3-TR isoforms in skeletal muscle

**Fig. 4:**
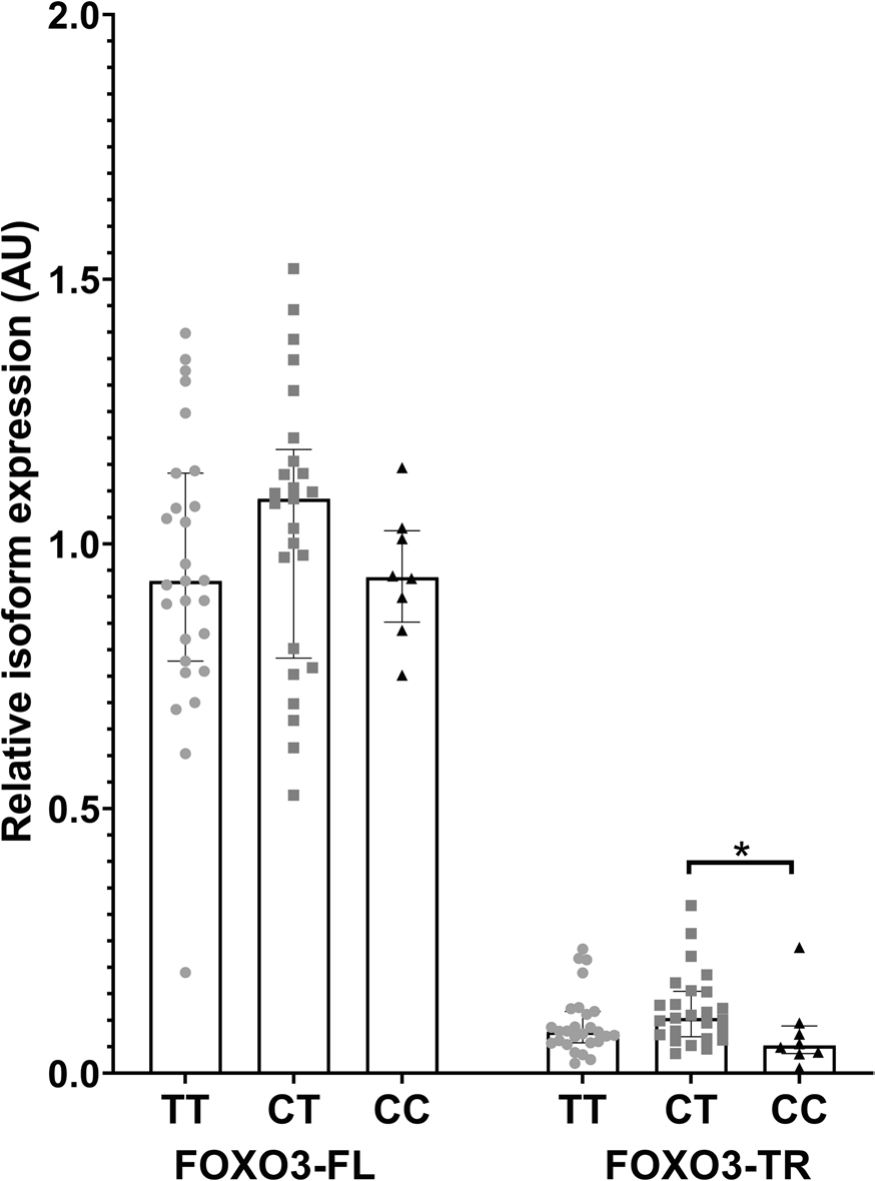
Relationship between *FOXO3* genotype and *FOXO3* isoform expression in skeletal muscle RNA samples. The relationship between *FOXO3* full length and truncated isoform levels and genotype at rs13217795 is given for muscle samples originating from the SPADE, BIER, TIME and INDIA studies. Isoform identity and genotype are given on the *X* axis, and median relative expression in arbitrary units (AU) is given on the *Y* axis. Statistical significance of logged data is indicated by stars; *p ≤ 0.05, **p ≤ 0.01, ***p ≤ 0.001. Circles indicate major allele (T) homozygotes, squares indicate heterozygous samples (C/T) and triangles indicate minor allele (C) homozygotes. Error bars represent interquartile range

We measured levels of *FOXO3-FL* and *FOXO3-TR* isoforms in RNA from human skeletal muscle samples, and related these to genotype at rs13217795. In contrast to our findings in in peripheral blood, we detected no association of *FOXO3-FL* isoforms with genotype at rs13217795 in skeletal muscle (Fig. 4). *FOXO3-TR* isoforms were expressed in muscle, consistent with our findings in the tissue panel (Fig. 2). Furthermore, we determined that in the skeletal muscle of individuals carrying two copies of the longevity ‘C’ allele, the logged mean expression of the *FOXO3-TR* isoform was 0.07 (SD = 0.07) compared with 0.12 (SD = 0.06) in individuals heterozygous for rs13217795 (*p* = 0.013). Levels of *FOXO3-TR* isoforms also demonstrated a trend towards reduced expression in C/C individuals compared with individuals homozygous for the ‘T’ allele, although this was not statistically significant, probably due to the large spread of data in the ‘T/T’ individuals. Comparison of FOXO3-TR levels in individuals heterozygous for the C allele did not display a similar reduction relative to TT homozygotes; however, levels are if anything slightly elevated in heterozygotes. This may suggest that 2 copies of the C allele may be necessary to observe a reduction in level.

### The full length FOXO3-FL isoforms display an association with age in human peripheral blood

To determine whether the *FOXO3-FL* isoforms displayed any evidence of differential expression with age, we correlated *FOXO3-FL* expression in peripheral blood mRNA with participant age in individuals homozygous for the major (T/T) allele of rs13217795 by linear regression. We identified that as expected, *FOXO3-FL* isoform expression declined with age (beta coefficient =  − 0.64; SE = 0.003, *p* = 0.012; supplementary figure S2).

## Discussion

The *FOXO3* gene is one of 4 human homologues of the *C. elegans Daf-16* gene, which was amongst the first genes associated with lifespan in animal models [20]. *FOXO3* belongs to a gene family which encodes 4 transcription factors (*FOXO1, FOXO3, FOXO4* and *FOXO6*) which are conserved from nematodes to mammals [24]. The unique characteristics of FOXO’s place them perfectly to act as cellular sensors, to allow cells to adapt and respond to their internal and external environment. Accordingly, FOXO proteins have important roles in multiple fundamental processes in cells, such as in control of metabolism, cell division and differentiation status and response to cellular stress [17] and unsurprisingly, have particular importance in regulation of ageing and longevity [25]. Genetic variation in the *FOXO3* gene has been linked with extreme longevity in multiple human populations [26, 31], although the molecular basis for this remains to be elucidated. Here, we have characterised the expression profile alternatively expressed isoforms of the *FOXO3* gene across a panel of human tissues, mapped SNPs associated with extreme longevity in human populations to the regulatory regions of the *FOXO3* gene and identified that individuals carrying extreme longevity alleles at rs13217795 demonstrate elevated expression of full length *FOXO3* isoforms in peripheral blood and decreased expression of the truncated *FOXO3* isoform in skeletal muscle.

**Fig. 5:**
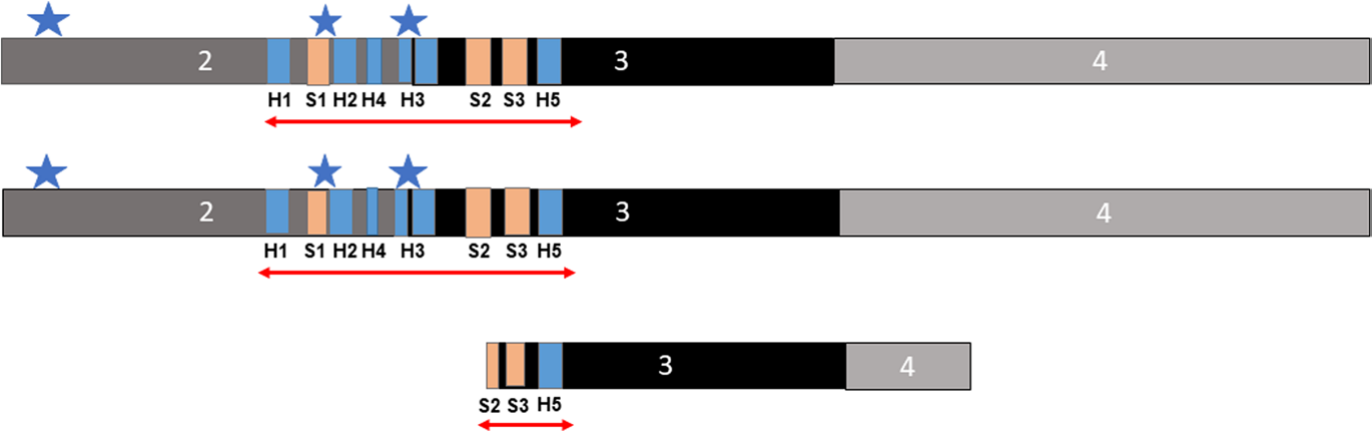
The functional domains of the FOXO3 protein in relation to isoform structure. The structure of the forkhead domain and position of functional posttranslational modifications within the FOXO3 gene relative to its isoform structure is given. The position of the forkhead domain is indicated by the red arrow. The five helices are given by blue boxes, and the spacer regions by orange boxes. The exon structure is given by black and grey boxes. Points of posttranslational modification at T32 and T179, and S207 are given by blue stars

FOXO proteins share a Forkhead DNA binding domain, which is approximately 100 bp long, and consists of three alpha helices and three beta pleated sheets flanked by looped and winged sections [13]. FOXO proteins bind as monomers to their downstream targets, and have been reported to be capable of both transactivation through direct binding to targets [10], or to gene repression, through DNA-independent indirect mechanisms [3, 11]. Transactivation or inhibitory activity can also be moderated by binding with cofactors such as SIRT1, CBP and p300 [27]. FOXOs are themselves regulated by posttranslational modifications such as phosphorylation, acetylation, methylation and ubiquitination [32]. The *FOXO3-FL* and *FOXO3-TR* isoforms demonstrate marked differences in their structure (Fig. 5). The full length isoforms contain the full length forkhead domain, whereas the truncated isoform contains only part of the S2 spacer, the S3 spacer and the 5th Helix H5, but is missing Helices H1 to H4 and spacer S1. The lack of a complete forkhead domain makes it unlikely that the truncated isoform would be able to efficiently bind DNA and regulate its downstream targets. The truncated *FOXO3* isoform is also lacking some points of posttranslational modification including phosphorylation sites at T32 and T179, and S207. T32 is one of three FOXO3 sites modified by AKT and SGK, that has been shown to create binding sites for the chaperone protein 14–3-3, which binds FOXO proteins in the nucleus and allows their active export [2]. This may mean that the truncated isoform also has altered ability to translocate between nucleus and cytoplasm, which has been shown to be a key factor in stress responses and determination of lifespan in nematodes [22, 28].

There may also be differences in miRNA regulation between isoforms, since the 3′ UTR of *FOXO3-TR* is truncated. The *FOXO3-FL* isoforms contain additional binding sites for isoforms contain additional binding sites for hsa-miR-29-3p, hsa-miR-155-5p, hsa-miR-9-5p, hsa-miR-27-3p, hsa-miR-122-5p, hsa-miR-142-3p.2 and hsa-miR-217, which are not present in *FOXO3-TR*. Although not all of these predicted binding sites have been functionally validated, inhibition of hsa-miR-155-5p has been shown to rejuvenate aged mesenchymal stem cells and enhances cardioprotection following infarction [16]. The FOXO3-TR isoform may therefore compete with the full-length transcripts for miRNAs that modulate (downregulate) *FOXO3* expression and, hence, the amount of protein. The longevity (minor) allele of rs13217795 and/or its proxies may functionally increase the amount of protein by serving as a miRNA sponge. There are also interactions with *circFOXO3*, a circular RNA, which we have previously described to be associated with cellular senescence and parental longevity in humans [15]. *CircFOXO3* has a role in cellular proliferation via sponging of miR-9-5p [23].

Our study is the first to characterise the alternatively expressed *FOXO3* isoforms in humans, and the first to report a shift in *FOXO3* isoform usage in human samples from different tissues in response to genotype at loci associated with extreme longevity. The tissue-specific effects we have noted also raise the possibility that different tissues may have different responses to genotype at such loci, particularly where the truncated isoform is predominant. Our work also has caveats; we have demonstrated these effects only at the level of the mRNA transcript, but others have documented the presence of a similar 5′ truncated FOXO3 protein isoform by Western blot in mouse [33]. We are also unable to precisely pinpoint the causal variant in or near *FOXO3* that underpins the effect we note, because of the tight LD between variants, but the most likely candidate is rs13217795 since it lies in a region of the *FOXO3* gene with characteristics of an alternative promoter. We cannot rule out an effect of its tightly linked proxy variant rs9400239, which lies in the alternatively expressed exon 1a of the *FOXO3* gene. Exon 1a is a non-coding exon, so will not alter the coding sequence of the truncated isoform, but it is possible that it confers some alteration in regulatory potential. Previous work has suggested that some of the longevity–associated SNPs at the *FOXO3* locus may act as a cis-regulatory unit that influences the expression level of neighbouring genes [7]. It is possible therefore that the longevity effect is due to both the potential alteration in overall FOXO3 activity brought by the change in expression of *FOXO3* isoforms, but also the changes in the expression of other genes that are influenced by FOXO3.

We propose here a potential explanation for the association reported in multiple human populations between genetic variation in intron 2 of the *FOXO3* gene and extreme longevity. We suggest that rs13217795, or one of its tightly-linked proxies, is capable of bringing about an upregulation of full-length *FOXO3* isoforms in some tissues, with downregulation of truncated forms of *FOXO3* being associated with inheritance of two copies of the longevity allele in others, most notably skeletal muscle. Some tissues may exhibit both phenomena, since we have observed differences between tissues in response. The truncated isoform of *FOXO3* is likely to have compromised DNA binding potential due to its interrupted forkhead domain, and may also have altered subcellular trafficking because of disrupted posttranslational modification. We propose that in individuals carrying one or more alleles of the rs13217795 variant (or a tightly linked proxy SNP), the shift towards full-length *FOXO3* isoforms and away from truncated transcripts brings about an enhancement of FOXO3 activity, with consequent effects on cellular processes involved in determination of lifespan.

## Supplementary Information

Below is the link to the electronic supplementary material.

Supplementary file1 (DOCX 1032 KB)

## References

[1] Anna A, Monika G (2018). Splicing mutations in human genetic disorders: examples, detection, and confirmation. J Appl Genet.

[2] Calnan DR, Brunet A (2008). The FoxO code. Oncogene.

[3] Chen CC (2010). FoxOs inhibit mTORC1 and activate Akt by inducing the expression of Sestrin3 and Rictor. Dev Cell.

[4] Davy PMC (2018). Minimal shortening of leukocyte telomere length across age groups in a cross-sectional study for carriers of a longevity-associated FOXO3 Allele. J Gerontol A Biol Sci Med Sci.

[5] Debey-Pascher S, Eggle D, Schultze JL (2009). RNA stabilization of peripheral blood and profiling by bead chip analysis. Methods Mol Biol.

[6] Dirks M, Wall B, Nilwik R, Weerts D, Verdijk L, van Loon L (2014). Skeletal muscle disuse atrophy is not attenuated by dietary protein supplementation in healthy, older men. J Nutr.

[7] Donlon TA (2017). FOXO3 longevity interactome on chromosome 6. Aging Cell.

[8] Dutta A, Henley W, Robine JM, Langa KM, Wallace RB, Melzer D (2013). Longer lived parents: protective associations with cancer incidence and overall mortality. J Gerontol A Biol Sci Med Sci.

[9] Dutta A, Henley W, Robine JM, Llewellyn D, Langa KM, Wallace RB, Melzer D. Aging children of long-lived parents experience slower cognitive decline. Alzheimers Dement. 2013;10:S315. 10.1016/j.jalz.2013.07.002. 10.1016/j.jalz.2013.07.002 24210527

[10] Eijkelenboom A, Burgering BM (2013). FOXOs: signalling integrators for homeostasis maintenance. Nat Rev Mol Cell Biol.

[11] Eijkelenboom A (2013). Genome-wide analysis of FOXO3 mediated transcription regulation through RNA polymerase II profiling. Mol Syst Biol.

[12] Fries JF (1980). Aging, natural death, and the compression of morbidity. N Engl J Med.

[13] Gajiwala KS, Burley SK (2000). Winged helix proteins. Curr Opin Struct Biol.

[14] Gallagher MD, Chen-Plotkin AS (2018). The post-GWAS era: from association to function. Am J Hum Genet.

[15] Haque S (2020). circRNAs expressed in human peripheral blood are associated with human aging phenotypes, cellular senescence and mouse lifespan. Geroscience.

[16] Hong Y (2020). miR-155–5p inhibition rejuvenates aged mesenchymal stem cells and enhances cardioprotection following infarction. Aging Cell.

[17] Huang H, Tindall DJ (2007). Dynamic FoxO transcription factors. J Cell Sci.

[18] Jameson TSO, et al. Reducing NF-kappa B signalling nutritionally is associated with expedited recovery of skeletal muscle function after damage. J Clin Endocrinol Metab. 2021;106:2057.10.1210/clinem/dgab106. 10.1210/clinem/dgab106 PMC8208676 33710344

[19] Kaplanis J (2018). Quantitative analysis of population-scale family trees with millions of relatives. Science.

[20] Kenyon C, Chang J, Gensch E, Rudner A, Tabtiang R (1993). A C. elegans mutant that lives twice as long as wild type. Nature.

[21] Kilroe SP (2020). Short-term muscle disuse induces a rapid and sustained decline in daily myofibrillar protein synthesis rates. Am J Physiol Endocrinol Metab.

[22] Lehtinen MK (2006). A conserved MST-FOXO signaling pathway mediates oxidative-stress responses and extends life span. Cell.

[23] Li Y, Qiao L, Zang Y, Ni W, Xu Z (2020). Circular RNA FOXO3 suppresses bladder cancer progression and metastasis by regulating MiR-9–5p/TGFBR2. Cancer Manag Res.

[24] Link W (2019). Introduction to FOXO biology methods. Mol Biol.

[25] Martins R, Lithgow GJ, Link W (2016). Long live FOXO: unraveling the role of FOXO proteins in aging and longevity. Aging Cell.

[26] Morris BJ, Willcox DC, Donlon TA, Willcox BJ (2015). FOXO3: a major gene for human longevity–a mini-review. Gerontology.

[27] Nasrin N (2000). DAF-16 recruits the CREB-binding protein coactivator complex to the insulin-like growth factor binding protein 1 promoter in HepG2 cells. Proc Natl Acad Sci U S A.

[28] Oh SW, Mukhopadhyay A, Svrzikapa N, Jiang F, Davis RJ, Tissenbaum HA (2005). JNK regulates lifespan in Caenorhabditis elegans by modulating nuclear translocation of forkhead transcription factor/DAF-16. Proc Natl Acad Sci U S A.

[29] Pavis GF (2021). Improved recovery from skeletal muscle damage is largely unexplained by myofibrillar protein synthesis or inflammatory and regenerative gene expression pathways. Am J Physiol Endocrinol Metab.

[30] Timmers PR, et al. Genomics of 1 million parent lifespans implicates novel pathways and common diseases and distinguishes survival chances. Elife. 2019;8:e39856. 10.7554/eLife.39856. 10.7554/eLife.39856 PMC6333444 30642433

[31] Willcox BJ (2008). FOXO3A genotype is strongly associated with human longevity. Proc Natl Acad Sci U S A.

[32] Xie Q, Chen J, Yuan Z (2012). Post-translational regulation of FOXO. Acta Biochim Biophys Sin (Shanghai).

[33] Xu C, Vitone GJ, Inoue K, Ng C, Zhao B (2019). Identification of a novel role for Foxo3 isoform2 in osteoclastic inhibition. J Immunol.

