# Correction to: Extreme longevity variants at the *FOXO3* locus may moderate *FOXO3* isoform levels

**DOI:** 10.1007/s11357-021-00465-4

**Published:** 2021-10-12

**Authors:** Ryan Frankum, Tom S. O. Jameson, Bridget A. Knight, Francis B. Stephens, Benjamin T. Wall, Timothy A. Donlon, Trevor Torigoe, Bradley J. Willcox, D. Craig Willcox, Richard C. Allsopp, Lorna W. Harries

**Affiliations:** 1 Institute of Biomedical and Clinical Science, University of Exeter Medical School, Barrack Road, Exeter, EX2 5DW UK; 2 College of Life and Environmental Sciences, University of Exeter, Exeter, UK; 3 NIHR Exeter Clinical Research Facility, Royal Devon and Exeter NHS Foundation Trust, Exeter, UK; 4 Honolulu Heart Program (HHP)/Honolulu-Asia Aging Study (HAAS), Department of Research, Kuakini Medical Center, Honolulu, HI 96817 USA; 5 Departments of Cell & Molecular Biology and Pathology, University of Hawaii, Honolulu, HI 96813 USA; 6 Institute for Biogenesis Research, Department of Anatomy, Biochemistry and Physiology, John A. Burns School of Medicine, University of Hawaii, Honolulu, HI USA; 7 Department of Geriatric Medicine, John A. Burns School of Medicine, University of Hawaii, Honolulu, HI 96817 USA; 8 Department of Research, Kuakini Medical Center, Honolulu, HI 96817 USA; 9 Okinawa International University, Okinawa, Japan

**Correction to: GeroScience**

10.1007/s11357-021-00431-0

The original version of this article contained errors in the affiliation and funding sections.

D. Craig Willcox’s affiliations should read ‘Department of Research, Kuakini Medical Center, Honolulu, HI 96817, USA’ and ‘Okinawa International University, Okinawa, Japan’. 

The authors would also like to acknowledge receipt of additional grant funding from the U.S. National Institute of General Medical Sciences (Grant P20GM103466).

