# Supplementary material for: Extreme longevity variants at the FOXO3 locus may moderate FOXO3 isoform levels
Source: GeroScience. 2021 Aug 26;44(2):1129–40. doi: 10.1007/s11357-021-00431-0 (PMC9135902; doi:10.1007/s11357-021-00431-0)
Supplement: Supplementary file 1 — Supplementary file1 (DOCX 1032 KB) [file 11357_2021_431_MOESM1_ESM.docx]

**Supplementary Figure S1: Representative standard curve analysis for assays to FOXO3:TR and FOXO3:FL isoforms**. A: FOXO3:FL. Standard curve produced from a serial 1:2 dilution of peripheral blood mRNA B: FOXO3-TR. Standard curve produced from a serial 1:2 dilution of skeletal muscle mRNA.


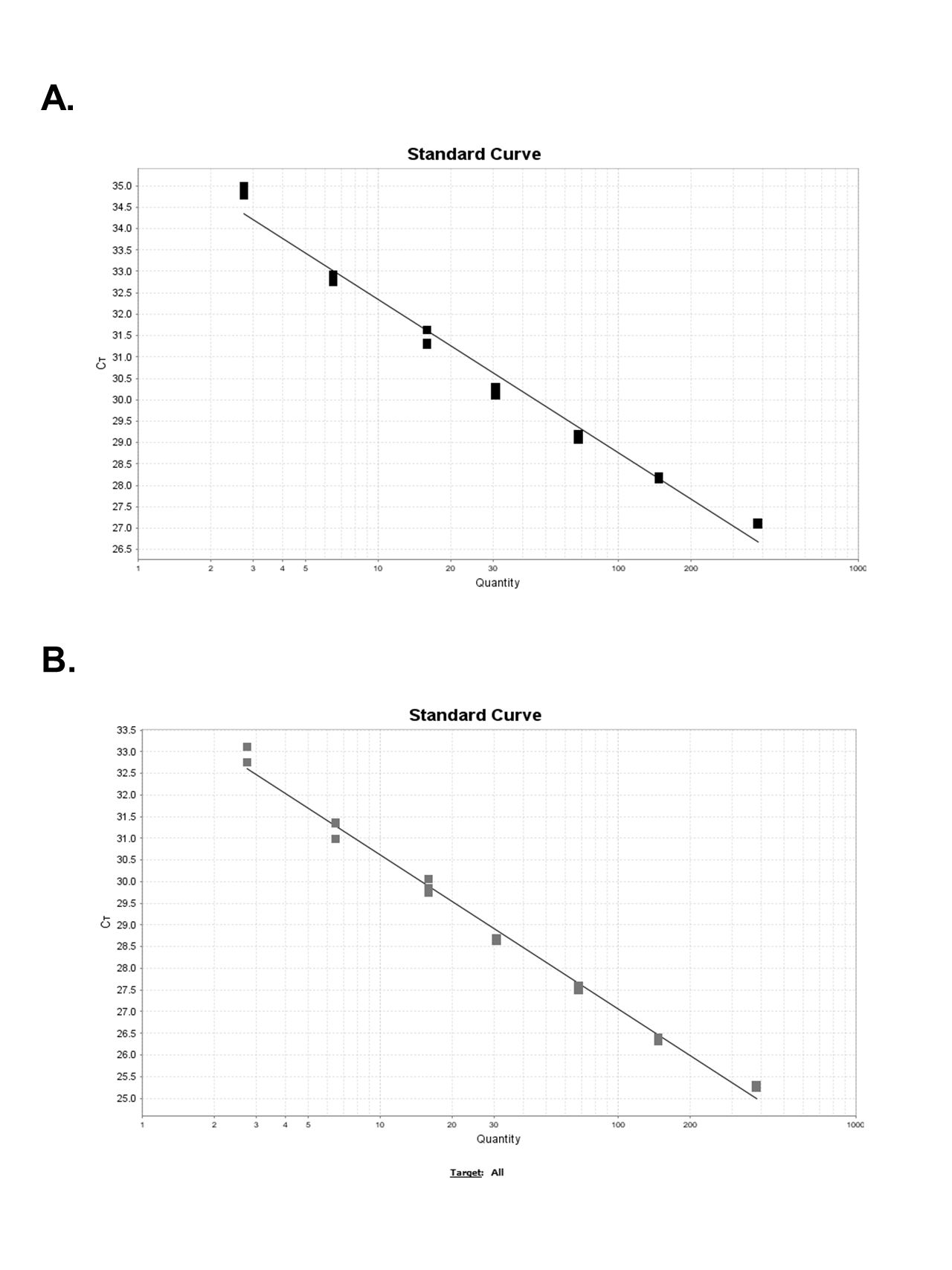


**Supplementary Figure S2: Correlation of expression with age**. The association between FOXO3-FL isoforms and participant age exclusively in 16 individuals homozygous for the major allele of rs13217795 is given in the graph below. Isoform expression is given on the Y axis and age is given on the X. The beta coefficient is -0.64, the standard error is 0.003 and the p value is 0.012. The regression line is indicated.
